# Cognition, attitude, practice toward health checkup and associated factors among urban residents in southwest China, Sichuan province, 2022: a community-based study

**DOI:** 10.1007/s10389-023-01883-8

**Published:** 2023-03-18

**Authors:** Min Du, Pingyang Li, Ling Tang, Min Xu, Xinzhu Chen, Huaicong Long

**Affiliations:** 1 North Sichuan Medical College, Nanchong, Sichuan People’s Republic of China; 2 Geriatric intensive care unit, Sichuan Academy of Medical Sciences & Sichuan Provincial People’s Hospital, School of Medicine, UESTC, Chengdu, Sichuan People’s Republic of China; 3 University of Electronic Science and Technology of China, Chengdu, China

**Keywords:** Health checkup, Cognition, Attitude, Practice

## Abstract

**Aim:**

Research on the health checkup status of urban residents in Southwest China is limited. In this study, we aimed to investigate the current status of health checkups and explore their influencing factors by analyzing the cognition, attitudes, and practices of urban residents in Southwest China.

**Methods:**

We sampled 1200 urban residents for a questionnaire survey. Statistical analysis was performed using SPSS 23, and logistic regression analysis was used to analyze the factors affecting cognition, attitudes, and practices regarding health checkups. A *P value* < 0.05 was used to identify variables significantly associated with the outcome variable.

**Results:**

Overall, 29% of the residents understood the importance of health checkups. The main ways urban residents acquire health-related knowledge are through the use of mobile media and medical staff health education. Only 40% of residents had undergone a regular checkup. Health self-assessment, economic reasons, and time are the factors that interfere with urban residents’ health checkups. Logistic regression analysis showed that occupation status, educational background, self-assessed health status, exercise status, and monthly income level were the common influencing factors of health checkup cognition and planning. Whether residents had participated in a medical checkup program was also related to sex and age.

**Conclusions:**

Urban residents in Southwest China generally had a high willingness to undergo physical examinations, but there were differences in knowledge and practice; at the same time, residents lacked understanding of respiratory assessments. Improving the health literacy of medical staff, strengthening urban residents’ health education, and enhancing the utilization rate of health checkups in urban residents are necessary and urgent.

## Introduction

Health checkup refers to the physical examination of subjects through medical means to evaluate the health status and to achieve the goals of disease prevention and early intervention (Virgini et al. 2015). The incidence of disease and preventable causes of death can be decreased by improving healthy behaviors and avoiding risk factors (such as regular exercise, consuming a balanced diet, smoking cessation, and abstinence from alcohol) (Moyer and U.S. Preventive Services Task Force 2012).

There has been controversy in the academic community regarding whether regular physical examinations should be carried out. Scholars who hold opposing opinions believe that although health checkups can help diagnose and treat diseases early, they do not affect the incidence of cardiovascular diseases and the mortality of cancer and other diseases (Gorbenko et al. 2017). Some scholars believe that the regular examination of healthy people wastes the time of examiners and doctors and increases the economic burden on examinees (Emanuel 2015). The general comprehensive screening method for the whole population may produce many false positives and negatives (Goodyear-Smith 2013), leading to more follow-up examinations, including unnecessary biopsies. However, most scholars believe that regular health examinations are critical and can help detect subclinical diseases and other health problems earlier, promote doctor–patient relationships, increase patients’ healthy educational opportunities, increase compliance with medication and lifestyle changes (Maciosek et al. 2010), and finally, reduce medical expenses. Compared with patients who did not undergo regular physical examinations, patients with regular physical examinations had significantly lower emergency department visit rates, hospitalization rates, and lengths of stay (Haruyama et al. 2017).

In most Western countries, regular health checkups are essential for clinical prevention (Steinkohl and Donner-Banzhoff 2014). Between 2002 and 2004, approximately 44 million adults in the United States received regular health checkups yearly (Mehrotra et al. 2007). Studies in Canada show that 10.5 million tests are performed yearly and that a “general physical checkup” is the second most common reason for medical visits after hypertension (Montreal 2009). According to the Market In-depth Research and Investment Strategy Forecast Report of China Physical Examination Hospital Industry, 2022–2027, the number of Chinese physical examination personnel increased from 450 million in 2016 to 640 million in 2020, with a compound annual growth rate of 9.2% (Zhongyan Puhua Group 2022). With the development of the social economy, health issues have received more attention. The construction of Healthy China has been elevated to the level of national strategy, which is a nationwide health improvement program in the new era that satisfies the common pursuit for a better life. While health checks are important, some residents still do not understand their importance. Some research data indicate that whether an individual receives a health checkup is often related to various factors, which usually affect each other. Occupation status, age, economic reasons, sex, education level, and access to medical services are the influencing factors in health examination decisions (Miller et al. 2014). However, there are specific differences in the situation of health examinations in different regions and populations.

Therefore, we conducted a questionnaire survey in four representative cities in Sichuan Province to determine the factors that affect residents’ health cognition, attitudes, and practices to improve their awareness of physical examinations and increase the rate of physical examinations. In addition, this study can provide a reference for health education and health policy formulation.

## Materials and methods

### Study design

**Fig. 1:**
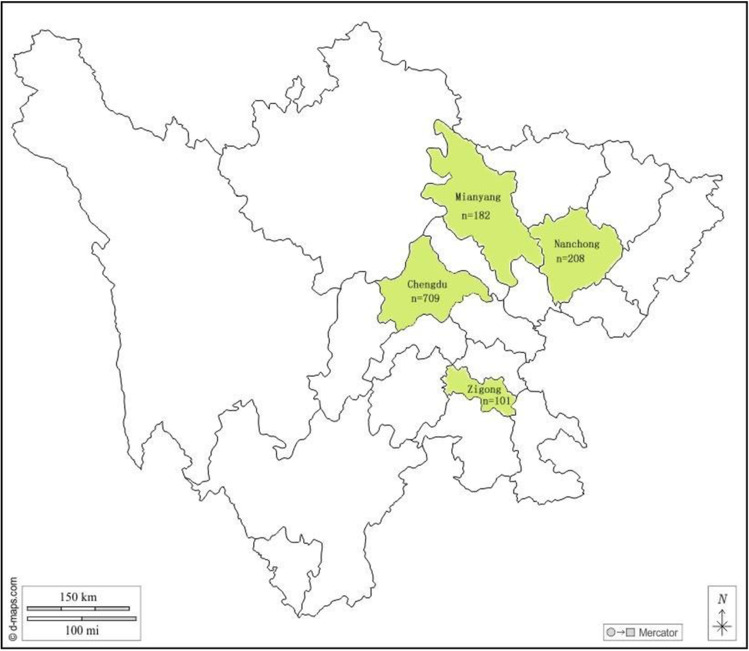
Study sites of urban residents in Sichuan

A cross-sectional study was conducted from January 2022 to April 2022. Sichuan Province is the most populous province in Southwest China; therefore, it is an excellent place to investigate. For this survey, four cities in different directions in Sichuan Province were selected. Details are as follows:

- Chengdu city (in western Sichuan), Zigong city (in southwestern Sichuan), Nanchong city (in northeastern Sichuan), and Mianyang (in northwestern Sichuan) were chosen as the study sites (see Fig. 1).
- Among the four cities, we chose to investigate counties with similar economic levels.
- Three streets were randomly selected from counties with similar economic levels for the questionnaire survey.
- One thousand two hundred residents from 12 streets were randomly sampled to complete the survey.

We selected the number of respondents according to the population in different regions, and we planned to have 1200 questionnaires (see Fig. 1 for details). This study was reviewed and approved by the Ethics Society of Sichuan Academy of Medical Sciences and Sichuan People’s Medical College (Ethics No. 515, 2021). Before starting the interview, all participants signed written informed consent forms.

### Participants

The inclusion criteria for participation were as follows: (a) participants aged between 20 and 75 years; (b) participants who lived in the survey area for at least one year, and (c) participants who voluntarily participated in the questionnaire. In addition, residents with cognitive impairment, those who were unable to complete the questionnaire, and those who were not of Chinese nationality were excluded.

### Questionnaire

Our questionnaire design was completed through a literature review and consultation with relevant experts. The questionnaire included five parts: (1) General information: sex, age, occupation status, educational background, personal monthly income, smoking status, drinking status, family disease history, self-assessment of health, and exercise habits; (2) cognition of health checkups: whether the participants understood the importance of health checkups, whether they had ever undergone a health checkup, and whether they understood the purpose of health checkups, and how to obtain health knowledge; (3) attitudes toward physical examination: whether they were willing to undergo a physical examination and whether they took the initiative to understand the relevant knowledge of physical examinations; (4) physical examination practices: frequency, institution, and payment method of health checkups; (5) knowledge about health screening for respiratory diseases.

### Definitions of relevant indicators

Smoking status was defined as follows: having smoked regularly, that is, smoking more than one cigarette per day or smoking more than four cigarettes per week for three consecutive months, with a cumulative number of cigarettes of more than 100 (Abnet et al. 2018). Drinking status was defined as follows: drinking ≥ one standard drink (approximately 10 g of pure alcohol) at least once in the past 30 days or drinking ≥1 standard drink at any time in the past(Witkiewitz et al. 2017). Exercise referred to the occupation status and the balance of domestic activities carried out in planned, targeted physical activity. Never exercising was defined as follows: never participating in any form of recreational physical activity in one’s spare time. Occasional exercise was defined as follows: *occasional exercise* 1–2 times per week for a duration of 10 min. Regular exercise was defined as taking part in leisure activities for at least 3 days per week, for at least 30 min per day, and with a moderate exercise intensity or above (Máderová et al. 2019).

### Quality control

Trained and qualified investigators conducted face-to-face interviews with each participant. They explained the research purpose clearly—participants completed the questionnaire anonymously, which were received by on-site investigators. We used two-person data entry for quality control, and participants with incomplete data were excluded.

### Statistical analysis

Descriptive statistical analysis was used to summarize the essential sociodemographic characteristics of the participants, and enumerative data are expressed as a constituent ratio (%). The chi-square test was used to screen the general demographic characteristics of cognition, intentions, and plans regarding physical examinations. An alpha level of less than 0.05 was statistically selected as a potential predictor for regression analysis. The dependent and independent variables were assigned values, and logistic regression analysis was used to determine the influencing factors of residents’ physical examination cognition, willingness, and plans. SPSS 23.0 was used for statistical analysis, and the level of statistical significance was set at 0.05.

## Results

### Sociodemographic characteristics of participants

**Fig. 2:**
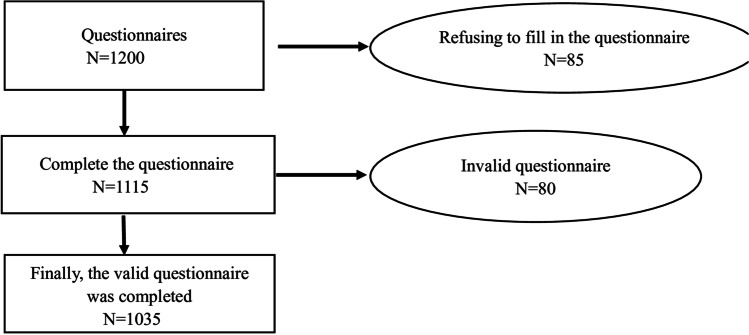
Data collection flowchart

In total, we received 1035 valid questionnaires. Eighty-five people refused to participate in the questionnaire, and 80 participants with incomplete questionnaires were excluded (see Fig. 2). More than half of the participants were female (61%); 27% were 20–29 years old, 22% were 30–39 years old, 19% were 40–49 years old, and 21% were 50–59 years old. Eleven percent of the participants were between 60 and 75 years old. Eleven percent of the participants had an undergraduate education and above, and 9% had a primary education and below. Seventy-four percent of the participants were married. Most were nonsmokers (81%) and nondrinkers (73%). Other demographic characteristics are shown in Table 1.

**Table 1:** Social demographic characteristics of the residents and influencing factors of health checkup

Variable	Classify	Frequency/n (%)	Meaning of physical examination				Willingness for health checkup				Health check-up plan			
			NO	Yes	*χ*^*2*^	*P value*	No	Yes	*χ*^*2*^	*P value*	No	Yes	*χ*^*2*^	*P value*
Gender	Male Female	407(39%) 628(61%)	294 445	113 183	0.229	0.632	30 36	377 592	1.111	0.292	203 256	204 372	10.111	0.001
Age	20~29 30~39 40~49 50~59 60~75	275(27%) 227(22%) 194(19%) 220(21%) 119(11%)	194 148 144 151 102	81 79 50 69 17	17.897	0.001	22 11 5 17 10	253 216 189 203 109	8.389	0.078	156 77 86 83 55	119 150 108 137 64	31.164	<0.001
Education	Primary school degree or below Junior high school Senior high school College degree University graduate or above	95(9%) 175(17%) 152(15%) 245(24%) 368(35%)	90 149 120 227 153	5 26 32 78 155	185.407	<0.001	11 12 14 15 14	84 163 138 230 354	10.524	0.032	65 100 73 72 87	30 75 79 173 281	110.485	<0.0001
Occupation	Unemployed Worker Professional technicians Clerks Freelancer Retire Others	128(13%) 92(9%) 139(13%) 114(11%) 93(9%) 77(7%) 392(38%)	100 72 63 68 68 65 30	28 20 76 46 25 12 90	71.432	<0.0001	11 9 40 5 6 5 26	117 83 19 109 87 72 366	217.044	<0.0001	84 42 41 25 41 20 205	44 50 98 89 52 57 187	79.779	<0.0001
Marital status	Unmarried Married Divorced Widowed	241(23%) 768(74%) 21(2%) 5(1%)	165 558 13 3	76 210 8 2	2.856	0.414	16 46 3 3	225 722 18 2	25.713	<0.0001	136 311 9 3	105 457 12 2	19.393	<0.0001
Smoking	No Yes	840(81%) 195(19%)	595 144	245 51	0.704	0.402	48 18	792 177	3.278	0.070	361 99	479 96	3.389	0.048
Alcohol drinking	No Yes	753(73%) 282(27%)	575 99	26596	21.787	<0.0001	49 17	704 265	0.079	0.779	329 130	424 152	0.482	0.488
Family history of the disease Relatives and friends with or without the disease	No	924(89%)	652	27240	2.049	0.152	58 8 55 11	566 103 741 228	0.503	0.478	416	508	1.585	0.208
	Yes	111(11%)	71								43	68		
	No	796(77%)	569	227 6							365	431		
	Yes	239(23%)	170	9	0.011	0.916			1.639	0.201	94	145	3.170	0.075
Chronic disease	No Yes	841(81%) 194(19%)	569 170	227 69	0.011	0.916	55 11	741 228	1.639	0.201	365 94	431 145	3.170	0.075
Self-rated health	Worse Good Excellent	60(6%) 590(57%) 385(37%)	52 447 240	8 143 145	27.832	<0.0001	6 35 25	54 555 360	1.523	0.467	35 277 147	25 313 238	12.304	0.002
Physical activity	Never Occasional Regular	244(23%) 555(54%) 236(23%)	190 398 151	54 157 85	11.384	0.003	27 24 15	217 531 221	12.901	0.002	132 234 93	112 321 143	12.808	0.002
Monthly income	<1000 CNY^a^	190(18%)	159	31	178.319	<0.0001	18	172	14.016	0.003	119	71	78.025	<0.0001
	1000~5000 CNY	439(42%)	339	100			29	410			222	217		
	5000~10000 CNY	260(25%)	178	82			14	246			90	170		
	>10000 CNY	146(15%) 3		113			22	124			29	117		

a: 1000 Chinese Yuan ≈ 147.9 US dollars as of 31 July 2022

### Cognition about health examinations

The results showed that 29% of the residents understood the importance of a health checkup (see Table 1); 450 people (44%) had undergone physical examinations, and 835 (80%) considered that the purpose of physical examinations was to detect and treat diseases early. Thirty-eight residents (4%) considered health checkups meaningless, and 162 (16%) believed that health checkups should be performed when they were not feeling well. Furthermore, we investigated how residents acquire knowledge of health examinations and the reasons that affect residents’ health examinations. The results showed that residents mainly acquired health checkup knowledge through books, newspapers, mobile media, and doctors’ advice. Among these, mobile media and medical staff information accounted for 32% and 22% of the residents, respectively, and 188 people (18%) did not have access to health knowledge. Reasons affecting residents’ health checkups included not understanding the necessity of health checkups, thinking that they were healthy, economic reasons, and a lack of time to go to the hospital. In the collected questionnaires, 24% and 21% of the respondents answered that they thought they were physically fit and did not have time to go to the hospital (see Table 2).

**Table 2:** Contents of the survey and influencing factors comparison

Survey content	Frequency/n (%)	Survey content	Response times/rate (%)
Received a checkup in the past		Access to health examination knowledge	
No	179 (17%)	No	188 (9%)
Part of the checkup	406 (39%)	Books and newspapers	392 (19%)
System health checkup	450 (44%)	Mobile phone, TV, and other media	663 (32%)
The purpose of health examinations		Education for medical workers	452 (22%)
There is no purpose	38 (4%)	Informed by relatives and friends	380 (17%)
To get checked out if you do not feel well	162 (16%)	Others	5 (1%)
Early detection of disease, early treatment	835 (80%)	Reasons that interfere with physical examinations	
Are you willing to go for a physical examination in one year?		Not understanding the need for a health check-up	170 (14%)
No	65 (6%)	I feel very healthy	293 (24%)
Yes	970 (94%)	Economic reasons	224 (19%)
Do you take the initiative to acquire knowledge related to a health examination?		Does not have time	255 (21%)
No	59 (6%)	Going to the hospital is a hassle	84 (7%)
Occasionally	581 (56%)	Others	178 (15%)
Attach great importance to	395 (38%)	Important systems	
Do you think a physical examination is necessary?		Cardiovascular system	486 (18%)
No	104 (10%)	Respiratory system	460 (17%)
Yes	931 (90%)	Digestive system	470 (17%)
What is your current status of health examinations in the last year?		Nervous system	337 (12%)
I got checked out when I was sick	355 (34%)	Endocrine system	51 (2%)
I did not go for a check-up when I was sick	14 (1%)	Reproductive system	378 (14%)
There is no set time for a physical	260 (25%)	All	419 (15%)
Periodic physical examination	406 (40%)	Others	147 (5%)
What is your frequency of health check-ups?		Do you think a routine chest check-up includes	
<1year	73 (13%)	Do not know	45 (3%)
Once a year	407 (71%)	X-ray	230 (13%)
1–2 years	65 (11%)	Chest computed tomography (CT)	733 (43%)
>2years	5 (5%)	Pulmonary function test	325 (19%)
What is your payment method for health check-ups?		Blood examination	296 (17%)
Self-paying	270 (47%)	Others	77 (5%)
Paid by workplace	306 (53%)		
Are you aware of chest CT scans?			
Don’t know	351 (34%)		
Radiation may be present, and chest CT is not the best option	207 (20%)		
Chest CT is a very accurate examination, and the radiation is acceptable	477 (46%)		
What do you think of lung function tests?			
Don’t know	178 (19%)		
Very important	840 (81%)		

### Residents’ attitudes toward health check-ups

The survey showed that 94% of the residents were willing to undergo a physical examination (see Table 1), 6% were not willing to undergo a physical examination, and 38% were very concerned about their knowledge of health examinations and were ready to take the initiative to understand it. Ten percent of the population did not think it necessary to undergo a health check-up. However, only 40% of the population underwent regular health checkups (see Table 2).

### The practices of residents regarding health check-ups

In our investigation, 56% of the residents had a physical examination plan, while 44% did not (see Table 1). Among the residents with a physical examination plan, once-a-year checkups accounted for 71%. Most residents (65%) go to a health checkup center, and 35% choose to go to an outpatient department for physical examinations. Regarding the cost of checkups, workplaces covered 53% of the employees’ health care costs, and 47% of the participants paid at their own expense. The survey showed that residents pay more attention to the cardiovascular, respiratory, and digestive systems. Forty-three percent of the residents thought a chest CT scan should be performed for routine lung examination, and the accuracy of a CT scan examination was better than that of a chest X-ray examination. Eighty-one percent of the residents believed that pulmonary function tests were necessary and were willing to undergo pulmonary function tests when needed. In comparison, only 7% of the residents were familiar with pulmonary function tests (see Table 2).

### Analysis of related influencing factors of health examinations among residents

In the survey of 1035 residents, 296 (29%) residents knew the meaning of a health check-up, 931 (94%) residents had the willingness to undergo a physical examination, and 576 (56%) residents had their physical examination plan. The chi-square test shows significant differences in age, education level, occupation status, drinking history, chronic diseases, self-assessed health, exercise, and monthly income between residents who knew and did not know the significance of physical examinations. There were significant differences in educational background, occupation status, marital status, physical exercise levels, and monthly income between residents who were and were not willing to undergo a physical examination. There were differences in gender, age, education, occupation status, marriage, smoking status, self-rated health, exercise level, and monthly income between residents with and without a physical examination plan (see Table 1).

### Regression analysis

Because other factors may confound the bivariate effect of the predictor on the dependent variable, the predictive effect of each potential predictor identified in the bivariate analysis was further examined using binary logistic regression analysis to adjust for the effect of other confounding variables. However, the results of binary logistic regression analysis showed that occupation level, educational background, health status, exercise status, and monthly income were the influencing factors of the concept of physical examinations. Professional and technical personnel with a college-level education and a monthly income of more than 10000 CNY and residents with a high self-rated health status were more aware of the importance of physical examinations. Marital status and exercise influenced the participants’ willingness to undergo a physical examination. Sex, age, occupation status, self-evaluation of health, educational level, exercise situation, and monthly income were influencing factors of whether the participants had a health checkup plan. Among women aged between 40 and 49 years, clerks and retirees with a college-level education and above, residents with high self-evaluated health, those performing occasional exercise, and those with a monthly income > 5000 CNY were more likely to have examination plans (see Table 3).

**Table 3:** Logistic regression analysis of the meaning, willingness, and plan of health check-up

Predictor	Reference category	Meaning of physical examination				Willingness for health check-up				Health check-up plan			
		*β*	*SE*	*P*	*RR* (95%CI)	*β*	*SE*	*P*	*RR* (95%CI)	*β*	*SE*	*P*	*RR* (95%CI)
Gender	Male									0.801	0.177	<0.001	2.228(1.576~3.149)
Age	20~29												
30~39		0.481	0.951	0.613	1.617(0.251~10.426)					0.634	0.250	0.011	1.885(1.155~3.075)
40~49		0.773	0.950	0.416	2.167(0.337~13.937)					0.448	0.272	0.100	1.565(0.918~2.668)
50~59		0.698	0.950	0.463	2.010(0.312~12.942)					0.649	0.278	0.020	1.913(1.110~3.299)
60~75		0.888	0.923	0.336	2.430(0.398~14.825)					0.522	0.371	0.159	1.686(0.176~1.808)
Education	Primary school	0.320	0.931	0.731	1.377(0.222~8.539)								
Junior high school		2.306	1.045	0.027	10.032(1.293~77.863)	0.170	0.499	0.733	1.186(0.446~3.150)	0.326	0.293	0.266	1.385(0.780~2.459)
Senior high school		2.853	1.042	0.006	17.337(2.249~133.646)	−0.063	0.512	0.901	0.938(0.344~2.561)	0.625	0.307	0.041	1.869(1.025~3.408)
College degree		3.650	1.026	<0.0001	38.460(5.150~287.213)	0.277	0.491	0.572	1.320(0.505~3.451)	0.969	0.291	0.001	2.636(1.491~4.660)
University graduate or above		4.056	1.027	<0.0001	57.766(7.715~432.529)	0.153	0.509	0.764	1.165(0.430~3.158)	0.798	0.297	0.007	2.222(1.241~3.977)
Occupation	Unemployed												
Worker		−0.130	0.419	0.757	0.878(0.387~1.996)	−0.498	0.560	0.374	0.607(0.203~1.822)	0.541	0.338	0.109	1.718(0.886~3.333)
Professional technicians	No	1.194	0.366	0.001	3.300(1.612~6.757)	0.158	0.613	0.797	1.171(0.352~3.897)	0.522	0.324	0.107	1.685(0.893~3.178)
Clerks	No	−0.055	0.386	0.886	0.946(0.444~2.017)	0.144	0.647	0.823	1.155(0.325~4.102)	1.151	0.352	0.001	3.163(1.586~6.307)
Freelancer	No	−0.257	0.397	0.518	0.774(0.355~1.686)	−0.138	0.602	0.819	0.871(0.268~2.835)	0.180	0.329	0.585	1.197(0.628~2.281)
Retire	Worse	−0.393	0.528	0.456	0.675(0.240~1.898)	0.179	0.687	0.794	1.196(0.311~4.596)	1.540	0.454	0.001	4.664(1.916~11.355)
Others	Never	−0.105	0.323	0.746	0.901(0.478~1.697)	0.082	0.441	0.853	1.085(0.457~2.577)	1.162	0.254	0.524	1.176(0.715~1.934)
Marital status	Unmarried												
Currently married						0.079	0.348	0.820	1.082(0.547~2.141)	0.286	0.239	0.231	1.331(0.834~2.127)
Divorced						−0.613	0.832	0.461	0.542(0.106~2.764)	−0.608	0.553	0.272	0.544(0.184~1.610)
Widowed						−2.190	1.054	0.038	0.112(0.014~0.884)	0.326	1.072	0.761	1.385(0.169~11.336)
Smoking	No									−0.169	0.207	0.415	0.845(0.563~1.267)
Alcohol drinking	No	−0.099	0.180	0.582	0.906(0.636~1.290)								
Chronic disease	No	0.591	0.239	0.013	1.805(1.130~2.884)								
Self-rated health	Worse												
Good		0.581	0.453	0.199	1.788(0.736~4.342)					0.435	0.310	0.161	1.544(0.841~2.838)
Excellent		0.994	0.464	0.032	2.703(1.089~6.711)					0.667	0.323	0.039	1.949(1.034~3.674)
Physical activity	Never												
Occasional		0.427	0.206	0.038	1.533(1.024~2.294)	1.062	0.299	<0.0001	2.893(1.609~5.199)	0.473	0.172	0.006	1.605(1.145~2.249)
Regular		1.053	0.253	<0.0001	2.865(1.746~4.702)	0.634	0.352	0.072	1.885(0.946~3.755)	0.489	0.221	0.027	1.631(1.059~2.513)
Income	<1000 CNY												
1000~5000 CNY		−0.062	0.300	0.836	0.940(0.521~1.693)	0.449	0.372	0.227	1.567(0.755~3.252)	0.109	0.219	0.617	1.116(0.726~1.713)
5000~10000 CNY		0.518	0.321	0.107	1.678(0.894~3.150)	0.598	0.451	0.185	1.818(0.751~4.399)	0.784	0.258	0.002	2.189(1.321~3.628)
>10000 CNY		1.025	0.351	0.004	2.788(1.400~5.550)	0.971	0.589	0.100	2.640(0.832~8.379)	1.663	0.318	<0.0001	5.275(2.828~9.839)

## Discussion

This study investigated the status of cognition, attitudes, and practices regarding physical examinations among residents in Southwest China and the possible influencing factors. The results showed that occupation status, educational background, self-rated health, physical activity, and monthly income were the common influencing factors of health examination cognition and physical examination plans. Moreover, whether residents had a physical examination plan was also related to gender and age. The willingness of residents in Southwest China was generally high. However, there were problems such as insufficient awareness of physical examinations, a low overall physical examination rate, and less knowledge of respiratory system-related examinations.

Our study showed that approximately 29% of the residents understood the importance of physical examinations and had a correct understanding of them. Nevertheless, this rate was lower than that in Beijing (52.5%) (Deng et al. 2017), Urumqi (53.9%) (Zhuang et al. 2019), and Shanghai (82%) (Xu et al. 2010). Forty-four percent of the residents had undergone physical examinations in the past two years. Most residents believed that the purpose of a physical examination was to detect problems and treat them as early as possible, which is similar to the research conclusion of Virgini et al. (2015). There are various ways for residents to obtain health knowledge, with information mainly through internet mobile media and medical staff education. This is similar to Yang et al.’s (2019) result. With the popularity of smartphones and the rapid development of information technology, the internet has become an essential medium that can deliver point-to-point health knowledge and guidance. E-mails, telephones, and other mediums can provide supplements for continuous health services propaganda. They can also reduce the risk of common chronic diseases, promote lifestyle changes, and improve blood glucose, lipid levels, blood pressure, and other health-related indicators (Yang et al. 2019). Information from relatives and friends is also a meaningful way to obtain health examination knowledge. Residents who received advice from their family or friends participated in health examinations at a rate approximately two times that of those who did not receive advice from their family or friends (Sugisawa and Sugihara 2011). Self-rated physical fitness, economic reasons, and time were the fundamental reasons that interfered with residents’ physical examinations, which is consistent with the conclusion of a British study (Harte et al. 2018).

In this survey, 40% of the residents underwent regular health check-ups, which is similar to the overall health check-up participation rate in Japan (Ministry of Health, Labour and Welfare 2016). Most residents thought that health check-ups were critical and were willing to accept them. However, there is a discrepancy between knowledge and practice. Ninety percent of the residents thought that physical examinations were critical, but only 40% underwent a regular physical examination. This situation may be related to a variety of factors. In the context of the COVID-19 pandemic, the health awareness of residents has improved. However, some residents choose to postpone or temporarily not undergo health examinations due to the influence of the pandemic (Leng et al. 2021). They believe that going out or visiting hospitals may increase their chance of infection. Economic concerns may also be a factor. Second, it is related to residents’ lack of correct understanding of health examinations and the insufficient propaganda and education of medical staff and institutions.

Sixty-five percent of the residents chose to go to the physical examination department or medical examination center for health examinations, and 35% had a health check-up when they visited the outpatient department. According to the survey, 53% of the residents’ medical examination expenses were paid by their workplace, while 47% paid out-of-pocket payments. Residents who underwent regular physical examinations had relatively good compliance (Labeit et al. 2013), paying more attention to their health. Residents who were employed were more likely to undergo regular health checks than those who were not. This may be due to the fees paid by their workplaces and the annual supervision. Currently, the state only provides part of the health examination for individuals 65 years of age or older free of charge, but there are problems such as incomplete inspection and few free items (Li 2019). Some residents chose to visit the outpatient department for health checkups, which may be associated with the coexistence of chronic diseases. There is more consultation time with clinicians in outpatient clinics, and outpatient doctors may know more about patients’ general conditions (doctors may make recommendations based on age, family history, and lifestyle choices). The patients can talk about their needs. Therefore, residents are more willing to cooperate with the physical examination arrangements of the outpatient doctors they are familiar with.

In this study, residents paid more attention to the cardiovascular, respiratory, and digestive systems, which may be related to the increased incidence of diseases of these systems in recent years (Andersson and Vasan 2018).

With the outbreak of COVID-19, people have become more aware of respiratory symptoms, but what residents know about respiratory examinations remains unclear. Therefore, we added the understanding of respiratory system-related examinations to the questionnaire. The survey revealed that 43% of the residents believed chest CT should be included in routine lung examinations, which may be more accurate than chest X-rays. While most residents know lung function tests are essential, few residents have had one performed in the past. This study showed that residents were willing to perform pulmonary function tests if needed, but little about pulmonary function-related knowledge was known. A Chinese survey based on 9130 COPD patients showed that between 2014 and 2015, the pulmonary function test rate of COPD patients aged 40 years or older was only 5.9%, which was very low. This may be related to the lack of awareness among medical personnel about the use of lung function in diagnosing and treating respiratory-related diseases. At the same time, patients also lacked knowledge of lung function (Lv et al. 2020).

Lung cancer is one of the malignant tumors with the highest morbidity and mortality in the world. Among malignant tumors, it ranks first in the morbidity and mortality of all malignant tumors in China (Zheng and Sun 2015). Due to the lack of apparent symptoms in early-stage patients, approximately 2/3 of patients are in the middle and late stages when they are diagnosed, while early low-dose CT can screen 85% of stage I lung cancers. Low-dose CT screening is a better method to actively detect early lung cancer actively. The results of the National Lung Screening Trial (NLST) in the United States showed that low-dose CT screening could reduce the mortality rate of lung cancer by 20% (Henschke et al. 2001). Studies in China have shown that the detection rate of low-dose CT primary lung cancer screening is 0.2–2.7%, and 77–100% of the detected lung cancers are stage I lung cancers (Chen et al. 2021). Therefore, early diagnosis and intervention are the keys to improving the survival rate and reducing the lung cancer mortality rate. Regular physical examination can achieve the early diagnosis of lung cancer.

In summary, educational background, occupation status, chronic diseases, self-rated health, exercise habits, and monthly income influenced physical examination awareness—the higher the educational background was, the better the understanding of physical examination-related knowledge. Compared with residents without chronic diseases, residents with chronic diseases were more aware of health checkup-related knowledge. Excellent self-rated physical conditions, regular exercise, and a monthly income of more than 10,000 yuan were all protective factors for knowledge of health check-ups.

Residents who liked to exercise were more willing to participate in physical examinations than those who did not like to exercise. Women, residents aged 30–39 and 50–59 years, residents with a high school education and above, employees and retirees, residents with good self-rated health, and residents with exercise habits and an income of more than 5000 yuan were more likely to have physical examination plans. This was in line with previous studies (Dickman et al. 2017). Women and older residents were more likely to go for annual checkups. Women may be more concerned about their health than men (Lee et al. 2021), consistent with previous reports from the United States, Austria, and the United Kingdom (Miller et al. 2014; Labeit et al. 2013; Lee et al. 2021).

The limitations of this study are as follows: (1) This was a cross-sectional study, and we could not confirm any causal relationship; hence, further longitudinal observation is needed. (2) No comparison was made between urban and rural residents in Southwest China; future research may be needed. (3) This study was limited to Southwest China and cannot represent the other regions of China. (4) In this study, residents completed the questionnaire alone; therefore, some residents may have had subjective expectations when completing the questionnaires.

## Conclusions

In conclusion, we observed that urban residents in the Southwest region of China generally have a high willingness for physical examinations. However, the rate of medical examinations among them is inadequate. This contradiction is related to a variety of factors. There are some problems, such as a lack of understanding of health examinations and the separation of willingness and behavior; this implies that we have a long way to go. It is necessary not only to enhance the health literacy of residents but also to improve the health education consciousness of medical staff. At the same time, the government’s guidance on health awareness is critical.
